# GSK-3β Controls Osteogenesis through Regulating Runx2 Activity

**DOI:** 10.1371/journal.pone.0000837

**Published:** 2007-09-05

**Authors:** Fumitaka Kugimiya, Hiroshi Kawaguchi, Shinsuke Ohba, Naohiro Kawamura, Makoto Hirata, Hirotaka Chikuda, Yoshiaki Azuma, James R. Woodgett, Kozo Nakamura, Ung-il Chung

**Affiliations:** 1 Center for Disease Biology and Integrative Medicine, University of Tokyo, Tokyo, Japan; 2 Sensory and Motor System Medicine, Faculty of Medicine, University of Tokyo, Tokyo, Japan; 3 Teijin Institute for Biomedical Research, Tokyo, Japan; 4 Ontario Cancer Institute, Princess Margaret Hospital, Toronto, Canada; Max Planck Institute of Molecular Cell Biology and Genetics, Germany

## Abstract

Despite accumulated knowledge of various signalings regulating bone formation, the molecular network has not been clarified sufficiently to lead to clinical application. Here we show that heterozygous glycogen synthase kinase-3β (GSK-3β)-deficient mice displayed an increased bone formation due to an enhanced transcriptional activity of Runx2 by suppressing the inhibitory phosphorylation at a specific site. The cleidocranial dysplasia in heterozygous *Runx2*-deficient mice was significantly rescued by the genetic insufficiency of GSK-3β or the oral administration of lithium chloride, a selective inhibitor of GSK-3β. These results establish GSK-3β as a key attenuator of Runx2 activity in bone formation and as a potential molecular target for clinical treatment of bone catabolic disorders like cleidocranial dysplasia.

## Introduction

Bone formation is such a dynamic and intricate process that its perturbation leads to a variety of bone catabolic disorders including skeletal malformations and osteoporosis. Accumulated molecular evidence has revealed the involvements of a number of signalings in this process: Runx2, Wnt, insulin/phosphatidylinositol 3-kinase (PI3K)/Akt, bone morphogenetic proteins/Smads, hedgehog, Osterix, etc [1]. Among them, Runx2 is known to be essential for osteoblastic differentiation, because its null mutation in mice exhibited the complete lack of bone [2]–[4]. The heterozygous loss leads to cleidocranial dysplasia in both humans and mice, which is attributed to impaired bone formation [4]. Despite accumulated knowledge of these osteogenic siganling molecules, the interactions among them to form the molecular network of bone formation have not been clarified sufficiently to lead to epochal therapeutics to treat bone disorders like cleidocranial dysplasia.

Glycogen synthase kinase-3 (GSK-3) was originally identified as a serine/threonine kinase involved in the regulation of glycogen deposition. The enzyme which comprises two isoforms, GSK-3α and GSK-3β, has since been implicated in many different biological processes including developmental patterning and cell survival as a regulatory switch that integrates numerous signaling pathways [5]. Among them, GSK-3β is known to be a key negative regulator of canonical Wnt/β-catenin and PI3K/Akt signalings [6], both of which have been reported to induce bone formation [7]–[13]. To investigate the *in vivo* role of GSK-3β, the present study analyzed the skeletal phenotype of GSK-3β-deficient mice, and investigated the underlying molecular mechanism.

## Results

### Increased bone mass in heterozygous Gsk-3β-deficient mice

**Figure 1:**
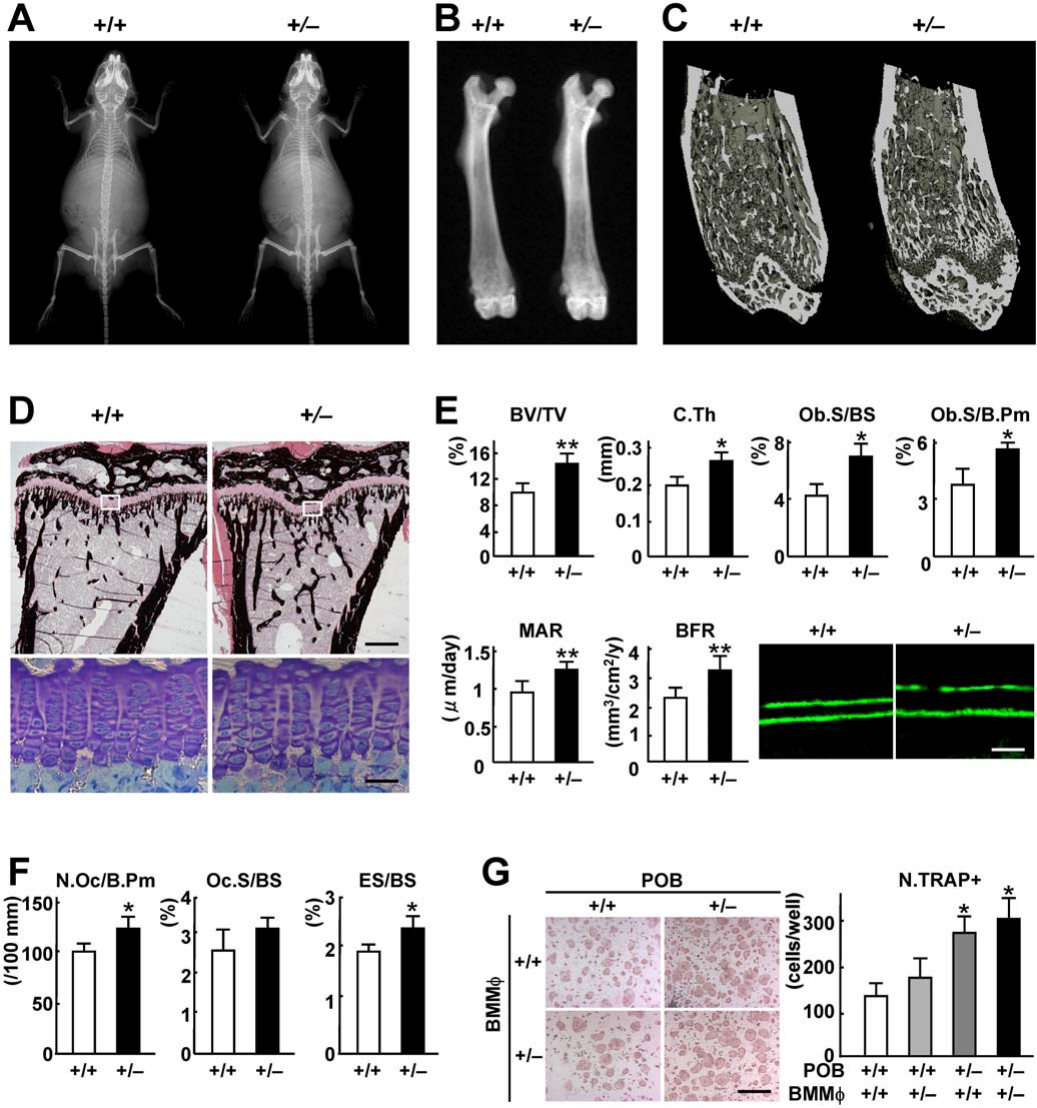
Increased bone mass due to the GSK-3β insufficiency by radiological and histological comparisons of *Gsk-3β^+/+^* and *Gsk-3β^+/–^* littermates at 12 weeks of age. (A) Plain X-ray of the whole body. (B) Plain X-ray of the entire femur. (C) 3D-CT image of the distal femur. (D) von Kossa staining of the proximal tibia (bar, 200 µm) and toluidine blue staining of the growth plate indicated by the inset box above (bar, 20 µm). (E) Histomorphometric analyses of bone volume and bone formation parameters in the proximal tibia. BV/TV, trabecular bone volume per tissue volume; C.Th, cortical thickness; Ob.S/BS, osteoblast surface per trabecular bone surface; Ob.S/B.Pm, osteoblast surface per trabecular bone perimeter; MAR, mineral apposition rate; BFR/BS, bone formation rate per trabescular bone surface. Lower right panel shows fluorescent micrographs of calcein-labeled mineralization fronts of the trabecular bones (bar, 10 µm). (F) Histomorphometric analyses of bone resorption parameters in the proximal tibia. N.Oc/B.Pm, number of osteoclasts per 100 mm of bone perimeter; Oc.S/BS, osteoclast surface per bone surface; ES/BS, eroded surface per bone surface. For (E) and (F), data are mean (bars)±SEM (error bars) of 10 mice per genotype. *P<0.05, **P<0.01 vs. *Gsk-3*β^+/+^. (G) Formation of TRAP-positive multinucleated osteoclasts by the co-culture of calvarial primary osteoblasts (POB) and bone marrow macrophages (BMMφ) derived from either *Gsk-3*β^+/+^ or *Gsk-3*β^+/–^ mice. Representative pictures (left; bar, 200 µm) and the number of osteoclasts expressed as mean (bars)±SEM (error bars) of 8 wells per group. *P<0.05 vs. *Gsk-3*β^+/+^ X *Gsk-3*β^+/+^.

To investigate the physiological role of GSK-3β in skeletal tissues, we examined the phenotype of *Gsk-3*β-deficient mice [14]. Although the homozygous *Gsk-3β-*deficient (*Gsk-3*β^–/–^) mice died in late embryogenesis due to severe liver dysfunction, heterozygous *Gsk-3β-*deficient (*Gsk-3*β^+/–^) mice developed and grew normally without disorders in major organs nor gross abnormality in the skeleton (Fig. 1A). However, the radiographs of the entire femurs and the three-dimensional computed tomography (3-D CT) of the distal femurs revealed that *Gsk-3*β^+/–^ mice showed an increased bone mass compared to the wild-type *Gsk-3*β^+/+^ littemates (Fig. 1B, C). Histological examination of the proximal tibiae confirmed the increases in both trabecular and cortical bones without abnormality in the growth plate, indicating that bone metabolism, not cartilage metabolism, was affected by the GSK-3β haploinsufficiency (Fig. 1D). In the bone histomorphometric analysis, the increased trabecular bone volume and cortical thickness in *Gsk-3*β^+/–^ mice were accompanied by significant increases in parameters of bone formation (Fig. 1E). Bone resorption parameters were also enhanced by the GSK-3β insufficiency, although weaker than bone formation parameters (Fig. 1F). Osteoclasts are known to be derived from hematopoietic cells and require cell-cell interactions with osteoblasts or stromal cells for differentiation. In the co-culture of calvarial primary osteoblasts and bone marrow macrophages (BMMφ), osteoclastogenesis was enhanced when osteoblasts, but not BMMφ, were derived from *Gsk-3*β^+/–^ mice (Fig. 1G), implicating that the enhanced bone resorption was due to the secondary effect of osteoblast dysfunction, but not the intrinsic defects of osteoclastic cells.

### Suppression of bone formation by *GSK-3*β in cultured osteoblasts

**Figure 2:**
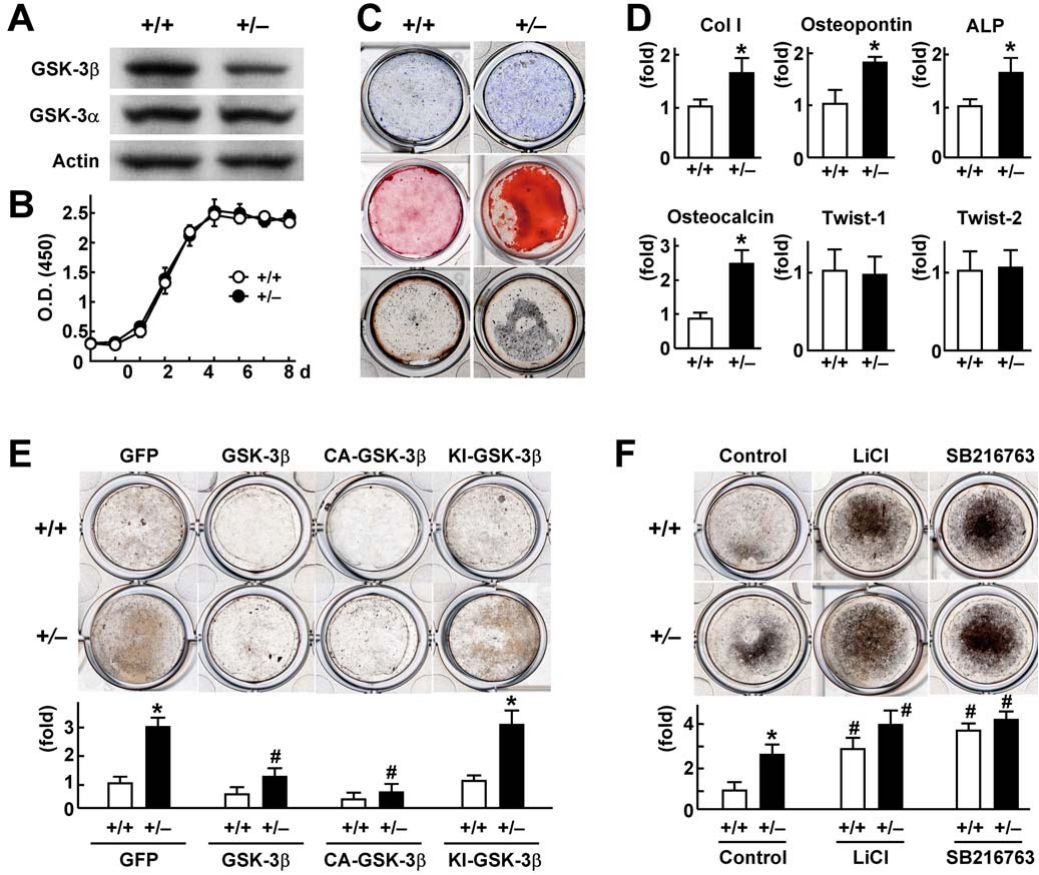
Suppression of bone formation by GSK-3β in cultured osteoblasts. (A) Expressions of GSK-3β and GSK-3α in calvarial osteoblasts of *Gsk-3*β^+/+^ and *Gsk-3*β^+/–^ littermates determined by immunoblot analysis with β-actin as a loading control. (B) Cell proliferation determined by the XTT assay in osteoblasts during 8 days of culture. Data are the mean (symbols)±SEM (error bars) of 6 dishes/genotype. (C) ALP (top), Alizarin red (middle), and von Kossa (bottom) stainings in osteoblasts cultured for 2 weeks. (D) mRNA levels of type I collagen (Col I), osteopontin, ALP, osteocalcin, Twist-1 and Twist-2, determined by real-time RT-PCR analysis in osteoblasts cultured for 2 weeks. Data are mean (bars)±SEM (error bars) of the relative amount compared to that of the *Gsk-3*β^+/+^ culture 6 wells per genotype. *P<0.01 vs. *Gsk-3*β^+/+^. (E) von Kossa staining (top) and osteocalcin mRNA level determined by real-time RT-PCR analysis (bottom) of osteoblasts transfected with the adenovirus expressing GFP, wild-type GSK-3β, constitutively active GSK-3β (CA-GSK-3β), or kinase-inactive GSK-3β (KI-GSK-3β), and cultured for 2 weeks. (F) von Kossa staining (top) and osteocalcin mRNA level (bottom) of osteoblasts cultured with and without lithium chloride (LiCl, 16 mM) or SB216763 (10 µM) for 2 weeks. For (E) and (F), the mRNA levels are mean (bars)±SEM (error bars) of the relative amount of mRNA compared to that of the control *Gsk-3*β^+/+^ culture of 6 wells per group. *P<0.01, significant stimulation by the genetic GSK-3β insufficiency. #*P<*0.01, significant effects by the adenoviral overexpression or the GSK-3β inhibitors.

To investigate the mechanism underlying the increased bone formation in *Gsk-3*β^+/–^ mice, we compared *ex vivo* cultures of calvarial osteoblasts derived from *Gsk-3*β^+/–^ mice with those from the *Gsk-3*β^+/+^ littermates. The GSK-3β protein level in the *Gsk-3*β^+/–^ osteoblasts was confirmed to be lower than that in the *Gsk-3*β^+/+^, while the GSK-3α level was comparable (Fig. 2A). Although cell proliferation was similar between the two genotypes (Fig. 2B), osteoblast differentiation and function determined by alkaline phosphatase (ALP), Alizarin red, and von Kossa stainings were enhanced in the *Gsk-3*β^+/–^ culture (Fig. 2C). Real-time RT-PCR analyses revealed that expressions of osteoblastic differentiation markers type I collagen (Col I), osteopontin, ALP, and osteocalcin were up-regulated by the GSK-3β insufficiency, whereas the differentiation markers of mesenchymal progenitors Twist-1 and Twist-2 were not affected [15] (Fig. 2D). Overexpression of the wild-type GSK-3β and constitutively active form of GSK-3β (CA-GSK-3β) via the adenoviral introduction significantly suppressed bone formation determined by the von Kossa staining and the osteocalcin mRNA level to similar levels in the two genotypes; however, overexpression of the kinase-inactive form of GSK-3β (KI-GSK-3β) did not affect it, indicating that kinase activity of GSK-3β is essential for its inhibitory action on bone formation (Fig. 2E). Contrarily, addition of lithium chloride or SB216763, selective inhibitors of GSK-3β, promoted bone formation in the *Gsk-3*β^+/+^ culture to the level similar to the *Gsk-3*β^+/–^ culture (Fig. 2F).

### Inactivation through phosphorylation of Runx2 by GSK-3β

**Figure 3:**
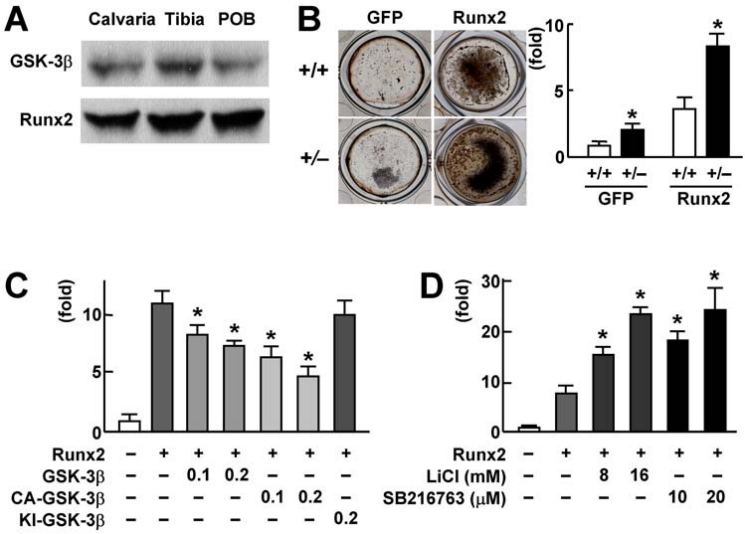
Suppression of Runx2 transcriptional activity by GSK-3β. (A) Expressions of GSK-3β and Runx2 determined by immunoblot analysis in mouse calvaria, tibia, and cultured calvarial primary osteoblasts (POB). (B) von Kossa staining (left) and osteocalcin mRNA level determined by real-time RT-PCR analysis (right) of *Gsk-3*β^+/+^ and *Gsk-3*β^+/–^ osteoblasts transfected with the adenovirus expressing GFP or Runx2, and cultured for 2 weeks. The mRNA levels are mean (bars)±SEM (error bars) of the relative amount of mRNA compared to that of the *Gsk-3*β^+/+^ with GFP culture of 6 wells per group. *P<0.01, significant stimulation by the Runx2 overexpression. (C) Luciferase reporter analysis of the effects of GSK-3β overexpression on the Runx2 transcriptional activity. HuH-7 cells were transfected with 1,050 OC-Luc alone or in combination with the plasmid expressing Runx2, and co-transfected with 0.1 or 0.2 µg plasmid expressing wild-type GSK-3β, CA-GSK-3β, or KI-GSK-3β, and cultured for 2 weeks. (D) Luciferase reporter analysis of the effects of GSK-3β inhibitors on the Runx2 transcriptional activity. HuH-7 cells were transfected with 1,050 OC-Luc alone or with the plasmid expressing Runx2, and cultured in the presence or absence of two doses of lithium chloride (LiCl) or SB21673 for 2 days. For (C) and (D), data are mean (bars)±SEM (error bars) of the relative activity compared to control culture of 6 wells per group. *P<0.01 vs. Runx2 alone.

We next examined the molecular mechanism underlying the GSK-3β inhibition of bone formation. In the two major osteogenic signalings in which GSK-3β is known to be involved, i.e., the canonical Wnt/β-catenin and the PI3K/Akt signalings [5]. A recent *in vivo* study showed that β-catenin hardly affected osteoblasts through a cell-autonomous mechanism [16]. Considering that the other signaling PI3K/Akt is related to Runx2 transactivation in its osteogenic action [17], we examined the involvement of Runx2 in the GSK-3β regulation of bone formation. We initially confirmed both GSK-3β and Runx2 expressions in the calvaria, tibia, and cultured osteoblasts (Fig. 3A). Bone formation determined by von Kossa staining and the osteocalcin mRNA level was enhanced by the Runx2 overexpression in both *Gsk-3β^+/+^* and *Gsk-3β^+/–^* calvarial osteoblast cultures (Fig. 3B). To examine the regulation of transcriptional activity of Runx2 by GSK-3β, a luciferase reporter gene construct containing a 1,050 bp osteocalcin gene fragment (1,050 OC-Luc) including the Runx2 binding sites was transfected into human hepatoma HuH-7 cells. The luciferase reporter analysis revealed that the Runx2-dependent transcription was suppressed by the co-expression of wild-type GSK-3β and CA-GSK-3β, but not by that of KI-GSK-3β (Fig. 3C), whereas it was enhanced by lithium chloride and SB216763 (Fig. 3D). Collectively, these data demonstrate that the kinase activity of GSK-3β suppresses the Runx2 transcriptional activity.

**Figure 4:**
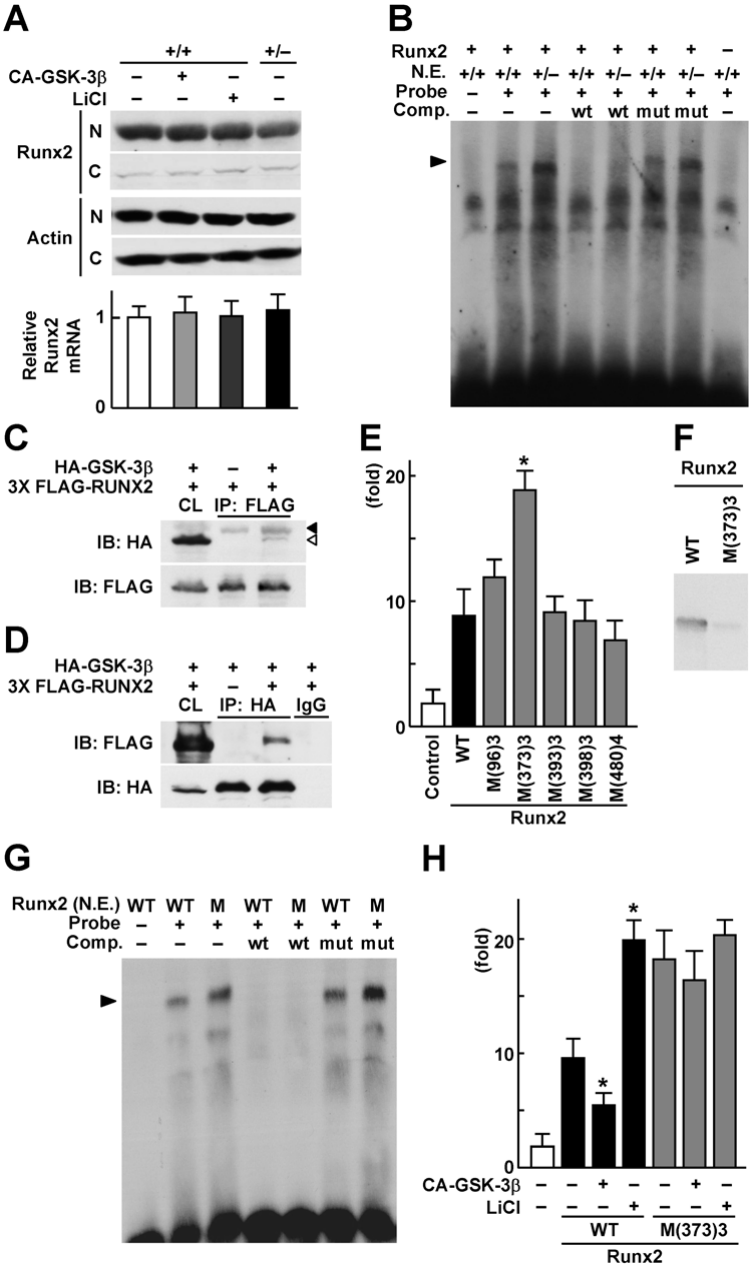
Inactivation through phosphorylation of Runx2 by GSK-3β. (A) Subcellular nuclear (N) and cytoplasmic (C) localizations of Runx2 by immunoblot analysis (top) and Runx2 mRNA level determined by real-time RT-PCR (bottom) in *Gsk-3*β^+/+^ and *Gsk-3*β^+/–^ calvarial osteoblasts overexpressing CA-GSK-3β or treated with LiCl, and cultured for 3 days. The mRNA levels are mean (bars)±SEM (error bars) of the relative amount compared to the control culture of 6 wells per group. (B) EMSA for specific binding (arrowheads) of a labeled OSE2 oligonucleotide probe with the nuclear extracts (N.E.) from *Gsk-3*β^+/+^ or *Gsk-3*β^+/–^ osteoblasts overexpressing Runx2. Cold competition (Comp.) was performed with 50-fold excess of unlabeled wild-type OSE2 probe (wt) and the mutated probe lacking the Runx2 binding sequence (mut). For controls, incubations without the probe (the 1st lane) and using nuclear extracts from osteoblasts without Runx2 transfection (the last lane) were performed. (C, D) Co-immunoprecipitation (co-IP) analysis of GSK-3b and Runx2. (C) Whole cell lysate (CL) and co-IP precipitant by anti-FLAG antibody-immobilized beads were immunoblotted with either anti-HA tag or anti FLAG tag antibodies. Filled arrowhead indicates non-specific band, and blank arrowhead indicates specific band. (D) Whole cell lysate (CL) and co-IP precipitant by anti-HA tag antibody or IgG (as a negative control) were immunoblotted with either anti-FLAG tag or anti-HA tag antibodies. (E) Luciferase reporter analysis of the effects of Runx2 mutations at the five consensus sites for the phosphorylation by GSK-3β on the Runx2 transcriptional activity. Mutations were created by three to four amino acid replacements as follows; S92A-S96A-S100A [M(96)3], S369A-S373A-S377A [M(373)3], S389A-T393A-S397A [M(393)3], T394A-S398A-T402A [M(398)3], and T476A-T480A-S484A-S488A [M(480)4]. HuH-7 cells were transfected with 1,050 OC-Luc alone or in combination with the plasmids expressing wild-type Runx2 (WT) or the mutants above, then cultured for 2 days. Data are mean (bars)±SEM (error bars) of the relative activity compared to control of 6 wells per group. *P<0.01 vs. WT-Runx2. (F) *In vitro* kinase assay. WT-Runx2 and M(373)3-Runx2 proteins were extracted by immunoprecipitation of the overexpresssing HeLa cells, and were incubated with recombinant GSK-3β. Reaction products were analyzed by immunoblotting using an antibody to phosphoserine. (G) EMSA for specific binding (arrowheads) of a labeled OSE2 probe with the nuclear extracts (N.E.) from HeLa cells transfected with wild-type Runx2 (WT) and M(373)3 Runx2 (M). Cold competition (Comp.) was performed as above. (H) Luciferase reporter analysis of the effects of GSK-3β signaling on the Runx2 transcriptional activity induced by WT-Runx2 and M(373)3-Runx2. HuH-7 cells were transfected with 1,050 OC-Luc alone or in combination with the plasmid expressing WT-Runx2 or M(373)3-Runx2 in the presence or absence of CA-GSK-3β overexpression or LiCl, then cultured for 2 days. Data are mean (bars)±SEM (error bars) of the relative activity compared to control of 6 wells per group. *P<0.01, significant effect of CA-GSK-3β overexpression or LiCl.

To further investigate how GSK-3β is involved in the Runx2 activity, we examined the effects of CA-GSK-3β overexpression, lithium chloride treatment, and the genetic GSK-3β insufficiency on the expression and subcellular localization of Runx2, and found that none altered either of them (Fig. 4A). We then transfected *Gsk-3*β^+/+^ and *Gsk-3*β^+/–^ osteoblasts with Runx2, and compared the binding of the nuclear extracts with the oligonucleotide probe of the Runx2 binding sequence, osteoblast-specific *cis*-acting element 2 (OSE2) of the mouse *osteocalcin* gene promoter [18], by electrophoretic mobility shift assay (EMSA). We found a complex that was confirmed to represent the Runx2-OSE2 binding, since it diappeared by the addition of 50-fold excess of unlabeled wild-type OSE2 probe, but not by the mutated probe lacking the Runx2 binding sequence, and was undetectable when the nuclear extract from cells without Runx2 transfection was used (Fig. 4B). The specific complex was augmented by the *Gsk-3*β^+/–^ nuclear extracts as compared to that by the *Gsk-3*β^+/+^ extracts, indicating that GSK-3β attenuates the DNA binding activity of Runx2. We then investigated biochemical interactions between Runx2 and GSK-3β by co-immunoprecipitation assay, which showed the possible direct interaction between these two molecules (Fig. 4C, D). To learn the contribution of the phosphorylation of Runx2 by GSK-3β to the attenuation of Runx2 transcriptional activity, we generated phosphorylation-deficient mutants of Runx2 by creating three to four amino acid replacements at the five consensus sites for the phosphorylation by GSK-3β [5]: S92A-S96A-S100A, S369A-S373A-S377A, S389A-T393A-S397A, T394A-S398A-T402A, and T476A-T480A-S484A-S488A. The luciferase reporter analysis using the 1,050 OC-Luc-transfected HuH-7 cells revealed that the phosphorylation-deficient mutant at S369-S373-S377 enhanced the transcriptional activity, while mutations at the other four phosphorylation sites showed comparable activity to the wild-type Runx2, indicating that the specific phosphorylation at S369-S373-S377 suppresses the Runx2 activity (Fig. 4E). *In vitro* kinase assay confirmed that the Runx2 phosphorylation by GSK-3β was reduced by the S369-S373-S377 mutation (Fig. 4F). When we compared the DNA binding of nuclear extracts from HeLa cells transfected with wild-type and the S369-S373-S377 mutant Runx2 by EMSA, the mutation enhanced the specific Runx2-DNA binding (Fig. 4G). Finally, the luciferase reporter analysis disclosed that the regulations of Runx2-dependent transcription by gain- and loss-of-functions of GSK-3β, i.e., suppression by CA-GSK-3β overexpression and enhancement by lithium chloride, were cancelled by the S369-S373-S377 mutation (Fig. 4H). These lines of results demonstrate that the phosphorylation of Runx2 at S369-S373-S377 by GSK-3β attenuates the transcriptional activity of Runx2, leading to the suppression of bone formation.

### Rescue of cleidocranial dysplasia by suppressing GSK-3β

**Figure 5:**
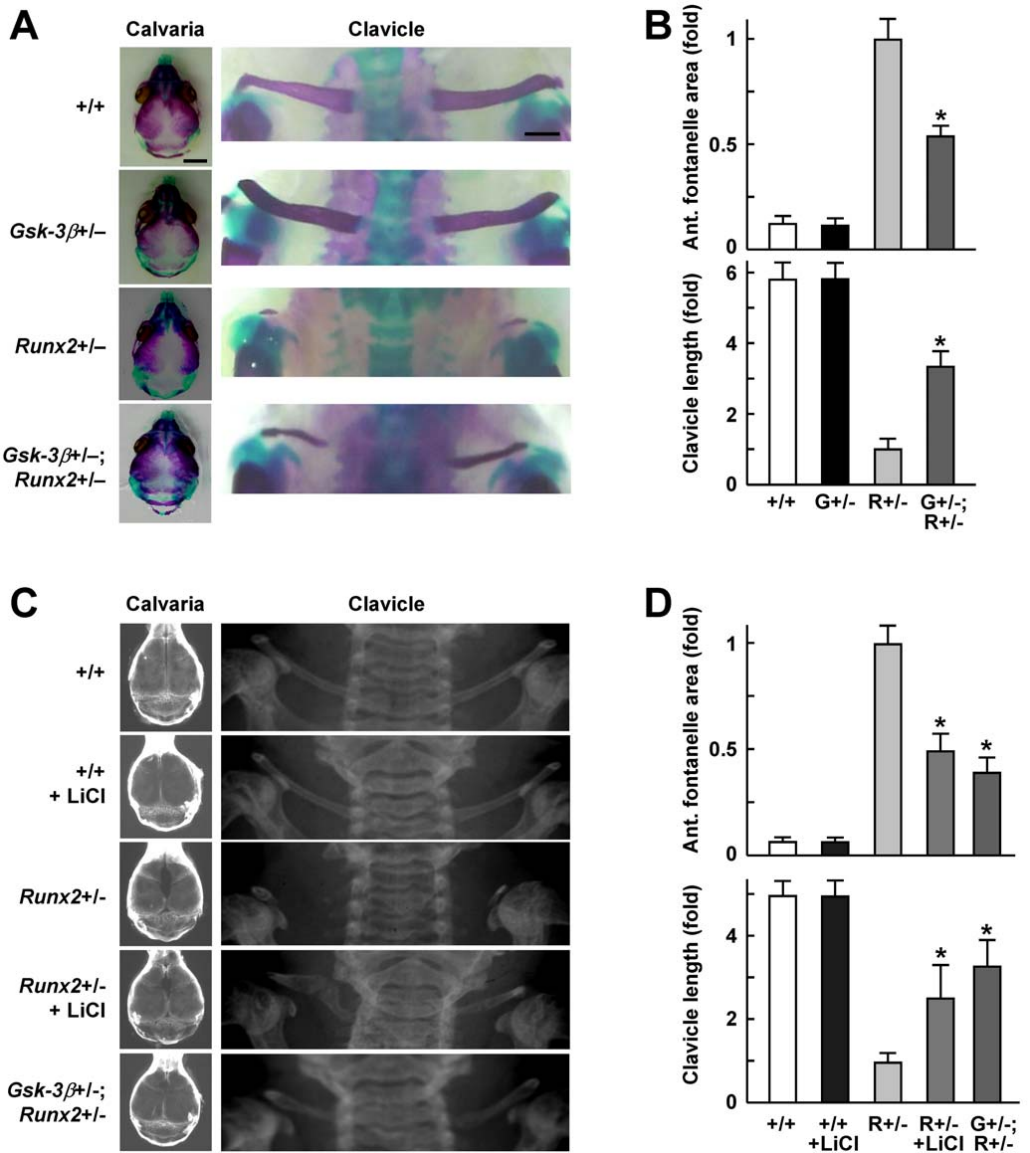
Genetic and pharmacological rescue of cleidocranial dysplasia by suppressing GSK-3β. (A) Calvarias and clavicles of *Gsk-3*β^+/+^, *Gsk-3*β^+/–^, *Runx2* ^+/–^, and *Gsk-3*β^+/–^ *; Runx2* ^+/–^ neonates (0-day) stained with Alizarin red and Alcian blue (bars, 3 mm for calvaria and 0.5 mm for clavicle). (B) Quantitative analyses using the NIH image of the anterior fontanelle area and the clavicle length of the four genotypes. (C) Plain radiographs at 3 weeks of age of the skulls and clavicles of *Gsk-3*β^+/+^ with and without LiCl administration from E7.5 to 3 weeks after birth, *Runx2* ^+/–^ with and without the LiCl administration, and *Gsk-3*β^+/–^ *; Runx2* ^+/–^ mice. (D) Quantitative analyses using the NIH image of the five groups. For (B) and (D), data are mean (bars)±SEM (error bars) of the relative amount compared to *Runx2* ^+/–^ of 6 mice per group. *P<0.01, significant rescue by genetic GSK-3β insufficiency or LiCl.

To investigate whether our *in vitro* finding on the molecular interaction between GSK-3β and Runx2 is reproducible *in vivo*, we crossed *Gsk-3β^+/−^* and *Runx2* ^+/−^ mice to generate the compound heterozygous deficient mice (*Gsk-3β^+/−^; Runx2^+/−^*), and analyzed the skeletal phenotypes of neonates. *Runx2* ^+/−^ mice, a model for human cleidocranial dysplasia, showed delayed closure of the fontanelle and hypoplasia of the clavicle due to impaired bone formation [3], [4], whereas *Gsk-3β^+/−^* mice had no such abnormalities. *Gsk-3β^+/−^; Runx2^+/−^* mice exhibited significant rescue of the both fontanelle and clavicle abnormalities of *Runx2^+/−^* mice (Fig. 5A, B).

Since this finding indicates the physiological interaction of GSK-3β with Runx2 function, we next examined a possible pharmacological intervention by lithium chloride that is reported to inhibit GSK-3β activity both *in vitro* and *in vivo* [19]-[21]. Because Runx2 is initially detected during embryogenesis at E9.5 in the notochord and at E10.5 in the mesoderm that is destined to develop to the shoulder bones [4], we administered lithium chloride to the embryos through pregnant and lactating dams from E7.5 to 3 weeks of age before weaning. We confirmed that the serum lithium concentrations of the mice treated with this regimen ranged from 0.66 to 0.70 mM, which falls on the lower side of the therapeutic range in humans (0.5–1.5 mM). Here again, the lithium chloride administration succeeded in restoring both fontanelle and clavicle abnormalities in the *Runx2^+/−^* mice, similarly to the genetic rescue in the *Gsk-3β^+/−^; Runx2^+/−^* mice (Fig. 5C, D), raising the possibility that pharmacological intervention such as lithium chloride administration may clinically be useful for preventing cleidocranial dysplasia.

## Discussion

### GSK-3β as a negative regulator of osteogenesis

The present *in vivo* and *in vitro* studies demonstrated that the suppression of GSK-3β in osteoblasts enhanced bone formation through a cell-autonomous mechanism. GSK-3β is a well-known negative regulator of the canonical Wnt/β-catenin signaling in that it induces proteasome degradation of β-catenin in the absence of the Wnt ligands. Binding of the Wnt ligands to the membrane frizzled receptor and low-density lipoprotein receptor-related protein 5 and 6 (LRP5/6) co-receptors inhibits GSK-3β, causing the stabilization of β-catenin which then translocates into the nucleus to activate the target genes like T cell factor (TCF) [22]. The Wnt signaling is known to be critical for maintaining bone mass, since gain- and loss- of functions of Lrp5 or Wnt10b positively correlated with bone mass in mice and humans [7]–[9], [23]. Furthermore, a recent study showing that lithium chloride increased bone formation even in Lrp5-deficient mice indicates that GSK-3β acts downstream of Lrp5 in the osteogenic action of the Wnt signaling [24]. Regarding the possibility of β-catenin being the target molecule of the GSK-3β action, recent reports on loss- and gain-of-functions of β-catenin have provided compelling evidence that β-catenin represents a differentiation switch of mesenchymal progenitors for inducing osteoblastic differentiation and suppressing chondrocytic differentiation at an early stage of skeletal development during embryogenesis [25]–[27]. In osteoblastic cells, however, β-catenin together with its target TCF proteins hardly affected their osteogenic function through a cell-autonomous mechanism, but regulated osteoblast expression of osteoprotegerin, a major inhibitor of osteoclast differentiation [16], indicating that osteoanabolic action of β-catenin is due to the decrease in osteoclastic bone resorption, but not due to the increase in bone formation. Hence, we hereby propose that the enhancement of bone formation by the GSK-3β suppression is mainly dependent on Runx2 rather than β-catenin. In addition, because the histomorphometric analyses of the *Gsk-3*β^+/–^ mice and the lithium chloride-treated mice [24] showed an increase in bone formation parameters, but not a decrease in bone resorption parameters, we assume that the bone anabolic action of the GSK-3β suppression is mediated by the Runx2 signaling for bone formation rather than the β-catenin signaling for bone resorption. The contribution and relationship between Runx2 and β-catenin as downstream signalings for the osteoanabolic action of the Wnt/Lrp5 should be further studied to elucidate the cellular and molecular network underlying the regulation of bone metabolism by the entire Wnt pathway.

### Attenuation of Runx2 by GSK-3β

A variety of hormones, cytokines and signaling molecules such as 1α,25(OH)_2_D_3_, tumor necrosis factor-α, fibroblast growth factor (FGF)-2, glucocorticoids, growth hormone, Akt, Stat1 & 3, Twist, Src/Yes, Dlx3, Msx2, PPARγ, and histone acetylases 3 & 4 have been reported to regulate Runx2 in its expression, subcellular localization, DNA binding, and transcriptional activity, although the mechanisms remain largely unknown [28]. The present study showed that GSK-3β inhibited the DNA binding and transcriptional activity through the S369-S373-S377 phosphorylation of the Runx protein. Regulation of Runx2 activity through its phosphorylation has been reported by phosphorylation-deficient mutagenesis at two conserved serines, S104 and S451 of the human *RUNX2* gene in distinct functional aspects [29]. The S104 phosphorylation is involved in the heterodimerization with the partner subunit PEBP2β, which enhances the transcriptional activity of RUNX2. On the other hand, the phosphorylation of S451 that resides within the C-terminal transcription inhibition domain of RUNX2 attenuates its transactivity. The consensus site T341 for the phosphorylation by PKA in the transactivation domain of mouse Runx2 is shown to be responsible for the induction of Runx2 transcriptional activity by parathyroid hormone (PTH) [30]. In addition, FGF-2 induces the Runx2 activity through phosphorylation of distinct consensus sites of ERK and PKC pathways [31]–[33]. Meanwhile, the present S369-S373-S377 is located in the negative regulatory region of DNA binding that masks the Runt domain and prevents it from binding to DNA [34]. Hence, the suppression of GSK-3β may relieve the GSK-3β-dependent phosphorylation of the negative regulatory region of Runx2, resulting in enhancement of DNA binding ability and transcriptional activity.

Insulin and insulin-like growth factor-I function as potent osteoanabolic agents [35], [36] via the activation of their common signaling molecules insulin receptor substrate (IRS)-1, IRS-2, and the subsequent PI3K/Akt. In fact, we and others previously reported that the loss-of-function mutation of *Irs-1*, *Irs-2, or* both *Akt1* and *Akt2* causes impairment of bone formation in mice [11], [12], [37]. As the target of this pathway, a recent study has shown that Akt enhanced transcriptional activity of Runx2 [17]. However, despite the fact that Akt is a serine-threonine kinase, the study failed to show the direct phosphorylation of Runx2 by Akt, and there was no consensus site for the phosphorylation by Akt in the Runx2 sequence. On the other hand, Akt is known to phosphorylate GSK-3β at Ser9, causing the inactivation [38]. We therefore speculate that the osteoanabolic action of the insulin/IRS/Akt pathway might also be mediated by the Runx2 phosphorylation by GSK-3β.

### GSK-3β as a potent therapeutic target for CCD and osteoporosis

The cleidocranial dysplasia phenotype by the Runx2 insufficiency was significantly rescued not only by the genetic suppression of GSK-3β, but also by the oral administration of lithium chloride. In addition, the GSK-3β insufficiency caused an increased bone mass in adult mice without other abnormalities. A previous study has revealed that the lithium chloride administration increased bone mass in normal C57BL/6 mice and osteoporosis model SAMP6 mice [24]. A recent report also showed that oral administration of LY603281-31-8, a small molecule inhibitor of GSK-3β and GSK-3α, increased bone formation, density and strength in an ovariectomized rat model [13] to the levels comparable to teriparatide (human PTH1-34), the only osteoanabolic drug that has recently been introduced into clinical practice for osteoporosis patients [39]. Taken together, these observations strongly suggest that the GSK-3β suppression may yield novel therapeutics to treat bone catabolic disorders like cleidocranial dysplasia and osteoporosis. Although characterization of small molecule inhibitors of GSK-3β is still underway, safety issues have not been reported at least for lithium chloride which is widely used by patients to treat bipolar disorder [40]. Hopefully prospective clinical trials on these drugs will be successful and generate epochal therapeutics for skeletal disorders.

## Materials and Methods

### Animals

Mice were maintained in a C57BL/6 background. In each experiment, male mice that were littermates generated from the intercross between *Gsk-3*β^+/+^ and *Gsk-3*β^+/–^ mice were compared. All experiments were performed according to the protocol approved by the Animal Care and Use Committee of the University of Tokyo.

### Radiological and histological analyses

Plain radiographs were taken using a soft X-ray apparatus. Micro CT scanning was performed using a composite X-ray analyzer (NS-ELEX Inc.), and cross-sectional tomograms of 10 µm thickness were reconstructed at 12×12 pixels into a 3-D feature by the volume-rending method. For von Kossa and toluidine blue stainings, samples were fixed with 70% ethanol, embedded in glycol methacrylate without decalcification, and sectioned in 3 µm slices. Histomorphometric analyses were performed in the secondary spongiosa (1.0 mm in length from 0.3 mm below the growth plate) of the proximal tibias using an image analyzer. For double labeling to analyze the dynamic bone remodeling, mice were injected subcutaneously with 8 mg/kgBW of calcein at 10 d and 3 d before sacrifice. Tartrate resistant acid phosphatase (TRAP)-positive osteoclasts were stained at pH 5.0 in the presence of L(+)-tartaric acid using naphthol AS-MX phosphate in N,N-dimethyl formamide as the substrate. Histomorphometric measurements were performed in eight optical fields, according to the ASBMR nomenclature report [41], and the averages were calculated per mouse. Alizarin red and alcian blue stainings of the whole mount skeleton of neonates were performed after they were fixed in 100% ethanol and transferred to acetone, as described previously [3]. The specimens were kept in 20% glycerol-1% KOH until the skeletons became clearly visible.

### Osteoclast formation assay

Osteoblasts were isolated from calvariae of neonatal mice, and bone marrow cells were collected from long bones of 8-week-old mice, as previously described [11], [12]. TRAP-positive multinucleated osteoclasts were generated by co-culturing osteoblasts (1×10^4^ cells/well) and bone marrow cells (5×10^5^ cells/well) derived from either *Gsk-3*β^+/+^ or *Gsk-3*β^+/–^ littermates in αMEM containing 10% FBS with 1α,25(OH)_2_D_3_ (10 nM) and prostaglandin E_2_ (100 nM) for 6 days. Cells positively stained for TRAP and containing more than three nuclei were counted as osteoclasts.

### Osteoblast cultures

Isolated calvaria osteoblasts were inoculated at a density of 2×10^5^ cells/well onto 24-well plates in αMEM containing 50 µg/ml ascorbic acid, 10 mM β-glycerophosphate, and ITS+1 liquid media supplement (Sigma-Aldrich) (osteogenic medium). For cell proliferation assay, cells were inoculated at 10^3^ cells per well in a 96-well plate and cultured for 8 days in the osteogenic medium with cell sampling every day. The proliferation of cells was quantified using an XTT {sodium 3,3-[(phenylamino) carbonyl]-3,4-tetrazolium-bis (4-methoxy-6-nitro) benzenesulfonic acid hydrate} Assay Kit (Roche). The absorbance of the product was quantified using a MTP-300 microplate reader (Corona Electric) read at 450 nm with reference wavelength 630 nm. The adenovirus vector carrying GFP, GSK-3β, CA- GSK-3β, KI- GSK-3β, or Runx2 gene was constructed using the Adeno-X Expression System (BD Biosciences), and was infected at 50 multiplicity of infection (MOI). The total MOI in each well was adjusted to be equal with the adenovirus encoding GFP. Two weeks after confluency, the total RNA was extracted, and the ALP, Alizarin red and von Kossa stainings were performed. For the ALP staining, cells were fixed in 70% ethanol and stained for 10 min with a solution containing 0.01% naphtol AS-MX phosphate disodium salt, 1% N, N-dimethyl-formamide, and 0.06% fast blue BB. For the Alizarin red staining, cells were fixed in 10% formalin/PBS and stained with 2% Alizarin red S (pH 4.0) solution. For the von Kossa staining, cells were fixed with 100% ethanol, stained with 5% silver nitrate solution under ultraviolet light, and incubated with 5% sodium thiosulfate solution (Wako).

### Real-time RT-PCR

The total RNA was extracted using an ISOGEN Kit (Wako) and an RNeasy Mini Kit (QIAGEN), and treated with DNaseI (QIAGEN), according to the manufacturers' instructions. One µg of RNA was reverse-transcribed with a Takara RNA PCR Kit (AMV) ver.2.1 (Takara) to generate single-stranded cDNA. PCR was performed with an ABI Prism 7000 Sequence Detection System (Applied Biosystems). Each PCR reaction consisted of 1 X QuantiTect SYBR Green PCR Master Mix (QIAGEN), 0.3 µM specific primers and 500 ng of cDNA. The mRNA copy number of a specific gene in total RNA was calculated using a standard curve generated by serially diluted plasmids containing PCR amplicon sequences, and normalized to the human or rodent total RNA (Applied Biosystems) with the mouse actin as an internal control. The standard plasmids were synthesized using a TOPO TA Cloning Kit (Invitrogen), according to manufacturer's instructions. All reactions were run in triplicate. Primer sequences are available upon request.

### Immunoblot and immunoprecipitation assays

Proteins were extracted with an M-PER or NE-PER Kit (Pierce Chemical), according to the manufacturer's instructions. Protein concentrations of cell lysates were measured using a Protein Assay Kit II (BIO-RAD). For immunoblot analysis, lysates were fractionated by SDS-PAGE with 4-20% Tris-Glycin gradient gel or 18% Tris-Glycin gel (Invitrogen) and transferred onto nitrocellulose membranes (BIO-RAD). After being blocked with 6% milk/TBS-T, membranes were incubated with an antibody to GSK-3α (Cell Signaling), to GSK-3β (Cell Signaling), to Runx2 (MBL), to HA tag (Upstate), to FLAG tag (Sigma-Aldrich), or to β-actin (Sigma-Aldrich). As secondary antibodies, HRP-conjugated antibodies to mouse IgG (Promega) and to rabbit IgG (Promega) were used. Immunoreactive bands were visualized with ECL Plus (Amersham), according to the manufacturer's instructions. Immunoprecipitation was performed using antibodies either noncovalently bound or conjugated to protein G-Sepharose (GIBCO). Equivalent amounts (20 µg) of cell lysate were immunoprecipitated with an antibodyb to Runx2 for 4 hours at 4°C. For co-immunoprecipitation (co-IP), cDNA encoding RUNX2 and GSK-3β genes were sub-cloned into p3XFLAG-CMV™ (Sigma-Aldrich) vector (3X FLAG-RUNX2) and pCMV-HA (Clontech) vector (HA-GSK-3β), respectively. Supernatant of centrifuged cell lysate, collected using RIPA lysis buffer (150 mM NaCl, 1.0% NP-40, 0.5% sodium deoxycholate, 0.1% sodium dodecyl sulfate, 50 mM Tris, pH 8.0) from 293T cells transfected with 3X FLAG-RUNX2 and/or HA-GSK-3β, was subjected to subsequent analysis. The co-IP complexes were recovered using EZview™ Red ANTI-FLAG® M2 Affinity Gel (Sigma-Aldrich) or ProFound™ HA Tag IP/Co-IP Kit (Pierce) according to the manufacturer's instruction.

### Luciferase reporter analysis and EMSA

Huh-7 cells were plated onto 24-well plates, and were transfected with 0.1 µg of the reporter constructs (1,050 OC-Luc) and 0.1 or 0.2 µg of the plasmids encoding wild-type or five kinds of phosphorylation-deficient mutants of Runx2, GSK-3β, CA-GSK-3β, or KI-GSK-3β using FuGENE6 (Roche Diagnostics), and cultured for 2 days. The amount of total DNA in each well was adjusted to be equal with the pEGFP vector. The luciferase assay was performed using a PicaGene Dual SeaPansy Luminescence Kit (Toyo Ink) and Lumat LB 9507 (Berthold Technologies). The level of luciferase activity was normalized to the level of Renilla luciferase activity. EMSA was performed using a DIG Gel Shift Kit (Roche), according to the manufacturer's instructions. In brief, nuclear extracts from *Gsk-3*β^+/+^ or *Gsk-3*β^+/–^ osteoblasts transfected with plasmid expressing wild-type Runx2, or HeLa cells transfected with wild-type or M(373)3 Runx2 were incubated with digoxigenin-labeled double-stranded oligo-dNT probes encoding the OSE2 sequence [18] and separated using non-denaturing PAGE, and the immunoreactivity for digoxigenin was visualized by chemiluminescence. For the competition experiment, 50-fold excess of unlabelled wild-type or the mutated OSE2 probe was added to the solution.

### In vitro kinase assay

Flag-wild-type Runx2 or Flag-M(373)3 Runx2 was prepared from the respective Runx2 overexpresssing HeLa cells by immunoprecipitation with an antibody to Flag. The immunoprecipitated protein and recombinant human GSK-3β (Upstate) were mixed in a reaction buffer (20 mM HEPES, 10 mM MgCl_2_, 10 mM MnCl_2_, 1 mM dithiothreitol, and 0.2 mM EDTA) with 1.6 mM ATP, and incubated at 30°C for 30 min. Reaction products were analyzed by immunoblotting using an antibody to phosphoserine (CHEMICON).

### Rescue of cleidocranial dysplasia by suppressing GSK-3β

*Runx2^+/−^* mice were kindly provided by T. Komori (Nagasaki University). For the genetic rescue, we crossed *Gsk-3β^+/−^* and *Runx2* ^+/−^ mice to generate the compound heterozygous deficient mice (*Gsk-3*β*^+/−^; Runx2^+/−^*), and compared the skeletal phenotypes of neonates with *Runx2* ^+/−^. For the pharmacological rescue, we administered lithium chloride from E7.5 to 3 weeks of age before weaning through the pregnant and lactating dams by feeding with pelleted chow containing 4 mg/kg lithium chloride along with 1.5% NaCl water as previously described [20], [21]. The mice were euthanized for radiological analyses at 3 weeks. The quantitative analysis of the area of the anterior fontanelles and the length of the clavicles on the histology and X-ray were performed using an NIH Image. The serum lithium concentration of the treated mice was measured by an atomic absorption spectrophotometer (Hitachi).

### Statistical analysis

All data are means±SEMs. Means of groups were compared by ANOVA and significance of differences was determined by *post-hoc* testing using Bonferroni's method.
